# Hypertension as a risk factor for periodontitis

**DOI:** 10.6026/973206300221717

**Published:** 2026-03-31

**Authors:** Jaideep Mahendra, Uma Subbiah, Aditippriya B.S, Muskan Bedi

**Affiliations:** 1 Department of Periodontics, Meenakshi Ammal Dental College and Hospital, Faculty of dentistry, Meenakshi Academy of Higher Education and Research, Chennai, Tamil Nadu, India; 2 Department of Medicine, Sri Ramachandra Medical College and Research Institute Chennai, Tamil Nadu, India

**Keywords:** Hypertension, periodontitis, cardiovascular risk, systemic inflammation

## Abstract

Hypertension has been increasingly linked to periodontitis, a chronic inflammatory disease of tooth-supporting tissues. Therefore, it is of interest to evaluate whether hypertension acts as an independent risk factor for periodontitis in 60 adults aged 35-65 years. Individuals with periodontitis showed significantly higher systolic and diastolic blood pressure and a greater prevalence of hypertension compared to controls. Hypertensive participants demonstrated an initial fourfold higher risk of periodontitis, though the association diminished after adjusting for age, obesity and diabetes. Data shows a connection between hypertension and periodontitis, but its independence may be moderated by metabolic comorbidities.

## Background:

Periodontitis is a prevalent chronic inflammatory disease affecting the supporting structures of teeth and has been consistently associated with systemic conditions such as cardiovascular disease [CVD] and hypertension [1, 2 and 3]. In India, periodontal disease affects approximately 51% of adults, with severe periodontitis reported in 19% of the population, and prevalence increasing significantly with advancing age [4, 5]. Periodontal infection elevates circulating inflammatory mediators such as interleukin-6, tumor necrosis factor-α, and C-reactive protein, contributing to endothelial dysfunction and arterial stiffness [3, 6, 7- 8]. Conversely, hypertension may aggravate periodontal tissue destruction by impairing microvascular function and tissue perfusion, thereby enhancing local inflammatory responses. However, the causal direction and independence of this association remain unclear [6]. Several epidemiological and cross-sectional studies have investigated the association between periodontitis and hypertension, reporting inconsistent findings due to confounding factors such as age, obesity, diabetes, and smoking habits [9, 3]. Very few studies have specifically evaluated hypertension as an independent risk factor for periodontitis after adjusting for the study variables, however the outcome has been inconsistent [9, 6]. Therefore, it is of interest to evaluate whether hypertension independently contributes to periodontitis risk or whether the association is influenced by shared metabolic and systemic factors.

## Materials and Methods:

## Study design and population:

A nested case-control study was designed. The patients with chronic periodontitis were screened for the present investigation based on the inclusion and exclusion criteria. Inclusion Criteria included the patients aged 35-65 years, both genders [male and female] diagnosed with chronic periodontitis, defined by the presence of at least 12 natural teeth and meeting clinical criteria for periodontitis. They had no history of periodontal treatment in the last six months and were willing to participate in the study. Controls were the individuals aged between 35-65 years, both genders [male and female] attending the same hospital without a diagnosis of periodontitis who had come master health checkup and no history of periodontal treatment in the last six months and were willing to participate in the study. Participants were excluded from the study if they had any of the following: [10] Subjects who had periodontal treatment in the past 6 months, with smoking/tobacco/alcohol habits, patients with carcinoma, sarcoidosis, immunosuppressive conditions, rheumatoid arthritis, and tuberculosis were excluded from the study [11]. Active infectious diseases including hepatitis, HIV, or tuberculosis; [1] were pregnant or breastfeeding; or [4] had used nonsteroidal anti-inflammatory drugs (NSAIDs) or antibiotics regularly within the past three months prior to evaluation. The study was approved by the Institutional Ethical Committee with Protocol no: MADC/IEC-I/3/2023B.

## Periodontal examination:

Periodontal assessment used a standardized protocol carried out by calibrated examiners using a UNC-15 probe. Clinical parameters such as probing depth, clinical attachment level and bleeding on probing were recorded in both the groups. Chronic periodontitis was characterized by slow to moderate progression, marked by clinical attachment loss (CAL), pocket formation, and/or gingival recession. Periodontitis severity was categorized based on CAL: slight (1-2 mm), moderate (3-4 mm), and severe (≥5 mm). The extent of the disease was classified as localized (≤30% of sites affected) or generalized (>30% of sites affected). Radiographic bone loss corresponds to CAL measurements, with slight cases showing up to 15% bone loss, moderate cases 16-30%, and severe cases exceeding 30% [5].

## Blood pressure assessment of systolic and diastolic assessment:

A trained individual used an Omron device M5-1 (HEM-757A-E) to take office blood pressure readings in accordance with a defined protocol, and each participant's readings were recorded in triplicate. In the half hour prior to their appointment, the patients were instructed not to smoke, exercise, or take any caffeine. After the patients were seated and relaxed for five minutes upon arrival, with their arms resting on a desk at the level of the right atrium and their backs resting on the chair, the measures were taken. Systolic blood pressure (SBP) and diastolic blood pressure (DBP) measurements were averaged to the closest value and utilized as continuous variables. This protocol aligns with the guidelines outlined by the American Heart Association (Pickering *et al.* 2005). Hypertension was defined as having systolic blood pressure ≥140, or diastolic blood pressure ≥90. Taking self-reported physician diagnosis or treatment for hypertension, blood pressure-lowering medication on medication inventory. Secondary outcomes of three-category severity of hypertension, was taken into consideration which was based on SBP and DBP cutoff points suggested by the Joint National Committee 7th report (Chobanian *et al.* 2003) (Stage 0 if SBP < 140 mmHg and DBP < 90 mmHg; Stage 1 if SBP 140 mmHg - 160 mmHg or DBP 90 mmHg - 100 mmHg; Stage 2 if SBP ≥ 160 mmHg or DBP ≥ 100 mmHg). The hypertensive SBP and DBP categories included those individuals who were under medication for hypertension [12].

## Study variables:

Socio-demographics information age, sex was recorded. Body mass index (BMI) was calculated as weight in kilograms divided by height in meters square. Body mass index (BMI) was coded as a categorical variable with BMI <18.5 kg/m^2^ as underweight; ≥ 18.5 kg/m^2^ and <25 kg/m^2^ as normal; ≥ 25 kg/m^2^ and <30 kg/m^2^ as overweight and ≥30 kg/m^2^ as obese. Anthropometric measurements (weight and height) were obtained using standard protocols and techniques. Diabetic parameters such as Fasting blood glucose, Glycated hemoglobin levels were recorded for all participants, were recorded for all participants in accordance with standard diagnostic criteria. These biomarkers are widely recognized for assessing glycemic status and long-term glucose control (American Diabetes Association, 2023; World Health Organization, 2011).

## Statistical analysis:

## Sample size calculation:

The sample size was estimated using G-power software based on previous studies indicating a prevalence of hypertensive patients with periodontitis at 66% and without periodontitis at 30%. With a power of 80% and a significance level of 5%, a total of 52 participants were required. To compensate for potential dropouts, the sample size was increased by 10%, resulting in 60 participants (30 Test group and 30 Control group). Data was analyzed using SPSS software (version 26, IBM, Chicago, USA). Continuous variables were expressed as mean ± standard deviation (SD) and analyzed using an independent sample t-test. Categorical variables were expressed as frequencies and percentages, with group comparisons performed using the chi-square test. Binary logistic regression was used to assess the association between hypertension and periodontitis, adjusting for confounders such as age, obesity, and diabetes. A p-value of <0.05 was considered statistically significant.

## Results:

Table 1 (see PDF) presents the socio-demographic characteristics of the study participants. The mean age of test group (54.03 ± 9.10 years) was significantly higher than that of control group (37.63 ± 10.26 years) (p = 0.00). The gender distribution was comparable between groups, with 14 males and 16 females in the control group and 13 males and 17 females in the test group (p = 0.39). The mean BMI did not show a statistically significant difference between test group (26.39 ± 4.39) and control group (25.70 ± 3.48) (p = 0.85). Obesity was observed in 20% of test group compared to 6.6% of control group, though the difference was not statistically significant (p = 0.27). Diabetes was present in 30% of test group, whereas none of the control group had diabetes, demonstrating a statistically significant association (p = 0.001). Table 2 (see PDF) outlines the association between hypertension and periodontitis. The mean systolic blood pressure (SBP) was significantly higher in test group (125.27 ± 19.80 mmHg) than in control group (113.93 ± 8.48 mmHg) (p = 0.001). Similarly, the mean diastolic blood pressure (DBP) was elevated in test group (78 ± 9.09 mmHg) compared to control group (74.53 ± 4.84 mmHg), with statistical significance (p = 0.05). Hypertension was more prevalent among test group (70%) than control group (36.6%) (p = 0.001), supporting a significant association between hypertension and periodontitis. Table 3 (see PDF) compares test and control group based on different stages of hypertension. Normal blood pressure was observed in 63.4% of controls and 30% of test group (p = 0.001). Pre-hypertension was present in 33.3% of test group and 36.6% of control group (p = 0.001). Stage 1 hypertension was found in 20% of test group, while none of the control group exhibited this condition (p = 0.001). Stage 2 hypertension was diagnosed in 16.7% of cases, with no occurrence in the control group (p = 0.001). These findings indicate a statistically significant trend of increasing hypertension severity among periodontitis in test group. Table 4 (see PDF) presents the results of binary logistic regression adjusting for confounders. The unadjusted odds ratio (OR) indicated that hypertensive individuals had a 4.07 times higher likelihood of being the test group compared to normotensive individuals (p < 0.001). After adjusting for age, the OR decreased to 1.55 but remained significant (p = 0.03). However, further adjustments for obesity (OR = 0.88) and diabetes (OR = 0.75) rendered hypertension a non-significant risk factor for periodontitis. These results suggest that obesity and diabetes may act as potential confounders, emphasizing the need for future studies with improved control of confounding variables. The study results indicate that individuals with periodontitis and hypertension were significantly older than control group, suggesting that age is a critical factor in the development of both periodontitis and hypertension. Our study observed a significant difference in age between test group [54.03 ± 9.10 years] and control group (37.63 ± 10.26 years) (p = 0.00), suggesting that periodontitis is more prevalent in older individuals (Table 1 - see PDF). This aligns with existing evidence that periodontal disease severity and hypertension prevalence increase with age due to cumulative bacterial exposure, immune dysregulation, vascular changes and impaired tissue repair mechanisms. Additionally, the association between hypertension and age was evident, with a higher prevalence of hypertension among older participants, reinforcing the role of aging as a contributing factor to both conditions [13]. This was consistent with the findings of Yang *et al.*, who reported that the association between periodontitis and hypertension was significant in individuals aged ≥50 years (p=0.018 for interaction), emphasizing the impact of aging on periodontitis [12]. In this study, gender distribution was comparable between groups (p = 0.39), indicating no significant influence of gender on the association between hypertension and periodontitis. Similarly, a cross-sectional study reported that no statistically significant differences were observed between males and females regarding the presence of periodontal disease, suggesting that gender alone may not be a determining factor in periodontal disease prevalence [14]. A secondary analysis of treated hypertensive adults aged ≥50 years showed that application of the 2021 European cardiovascular prevention guidelines markedly increased the proportion of patients classified as high/very high cardiovascular risk, with slightly lower hypertension control rates compared to previous standards, highlighting the need for stricter risk factor management [15]. Similarly, Giralt *et al.* stated that long-term outcomes and cardiovascular risks may be comparable between men and women; the presence of distinct sex-specific biological and hormonal influences underscores the need for personalized, gender-sensitive approaches in hypertension evaluation and management to ensure optimal and equitable cardiovascular care [16]. Larger gender-stratified investigations are needed to clarify the role of sex-related biological and behavioral factors in the periodontitis-hypertension relationship [15]. The mean BMI did not show a statistically significant difference between test group (26.39 ± 4.39) and control group (25.70 ± 3.48) (p = 0.85), suggesting that BMI alone may not be a decisive factor in periodontitis. (Table 1 - see PDF) Previous studies have shown conflicting findings regarding the association between obesity and periodontitis. While some research indicate a significant link between increased BMI and periodontal inflammation due to systemic inflammation and altered immune responses, others like Harris *et al.* found that BMI is not an independent risk factor for periodontitis when controlling for other metabolic parameters [17]. Furthermore, studies by Abu Shawish *et al.* (2022) highlight that visceral fat and metabolic syndrome components, rather than BMI alone, may have a stronger impact on periodontal disease progression. Obesity was more prevalent in the test group (20%) than in the control group (6.6%), though this difference was not statistically significant. This finding suggests a potential link between obesity and periodontitis, as obesity is associated with systemic inflammation, oxidative stress, and altered immune responses, all of which contribute to periodontal breakdown. The lack of statistical significance may be attributed to sample size limitations or other confounding factors. In future, research should focus on more specific obesity markers, such as waist-to-hip ratio or inflammatory biomarkers, to better understand the relationship between obesity and periodontitis [18]. The significant association between diabetes and periodontitis observed in this study is consistent with existing literature, reinforcing their well-documented bidirectional relationship. In our study, diabetes was present in 30% of test group, whereas none of the control group had diabetes, which was statistically significant (p = 0.001). This aligns with a systematic review by Rodrigues *et al.* who reported a higher prevalence of diabetes among periodontitis patients, linking hyperglycemia to impaired immune responses and increased oxidative stress [19]. Hasan *et al.* highlighted that diabetes increases the risk of periodontitis, while Stöhr *et al.* found a 26% increased risk of diabetes in individuals with periodontitis and a 24% increased risk of periodontitis in diabetics [20, 21]. These findings suggest a vicious cycle where chronic periodontal inflammation worsens glycemic control. This emphasizes the need for early detection and management of periodontitis in diabetic individuals. The significant association between hypertension and periodontitis was observed in this study. Mean SBP was significantly higher in test group (125.27 ± 19.80 mmHg) than in control group (113.93 ± 8.48 mmHg) (p = 0.001), while mean DBP was also elevated in test group (78 ± 9.09 mmHg) compared to control group (74.53 ± 4.84 mmHg) (p = 0.05). Hypertension was more prevalent among test group (70%) than control group (36.6%) (p = 0.001), reinforcing the link between periodontitis and elevated blood pressure. (Table 2 - see PDF) Moreover, the results showed an increase in the severity of hypertension among individuals with periodontitis, with a higher proportion exhibiting Stage 1 and Stage 2 hypertension compared to controls. (Table 3 - see PDF) This gradation suggests that the severity of periodontal inflammation may correlate with the progression of hypertensive status. This was in accordance with the findings of Zhan *et al.* who reported that hypertension prevalence and stage severity increased significantly with periodontitis severity in the Fourth National Oral Health Survey of China [8]. Muñoz Aguilera *et al.* [6]. Also found a significant association between periodontitis and elevated blood pressure. In his cross-sectional analysis of over 250 adults, individuals with periodontitis showed higher systolic and diastolic blood pressure levels, even after adjusting for confounders such as age, BMI, and smoking. He attributed this link to endothelial dysfunction and systemic inflammation triggered by periodontal disease, involving elevated inflammatory mediators that impair vascular health. A longitudinal study by Torrungruang *et al.* further confirmed that periodontitis patients with poor oral hygiene will be associated with increased hyoertension risk and systemic inflammation [7]. Similarly, a large case-control study reported that individuals with periodontitis had significantly higher systolic blood pressure and increased odds of hypertension, independent of traditional cardiovascular risk factors [6]. The underlying mechanisms linking these conditions include chronic inflammation, endothelial dysfunction, and microbial dysbiosis. Periodontitis induces systemic inflammatory responses, increasing pro-inflammatory cytokines like IL-6 and TNF-α, which contribute to vascular dysfunction and hypertension. Additionally, the periodontal pathogen load may lead to immune-mediated arterial changes, exacerbating blood pressure regulation [22]. Our findings, in conjunction with existing literature, underscore the importance of periodontal health in cardiovascular risk management. Given the strong association between hypertension and periodontitis, routine periodontal screening in hypertensive patients and vice versa could facilitate early intervention and better management of both conditions. Muñoz Aguilera *et al.* also advocated for integrated care strategies that bridge dentistry and medicine to address shared inflammatory pathways. Further analysis through binary logistic regression indicated that hypertension was a significant risk factor for periodontitis in unadjusted models (OR-4.07) and for adjusted model (OR-1.55) and for pre-hypertension, the unadjusted models showed OR -1.15. This was statistically significant (Table 4 - see PDF). However, after adjusting for confounding variables such as age, obesity, and diabetes, the association was no longer statistically significant. Our study's findings suggest that the initial association observed between hypertension and periodontitis may be influenced by shared metabolic and systemic factors such as age, obesity, and diabetes. This aligns with existing literature (Del Pinto *et al.* 2023; Larvin *et al.* 2022) indicating that the relationship between these two conditions is complex and potentially mediated by common risk factors [15, 23]. A cross-sectional survey from the Fourth National Oral Health Survey of China reported a significant association between periodontitis and hypertension, independent of age, gender, and smoking. However, the study also noted that hypertension prevalence increased with periodontitis severity, particularly among younger participants (35-44 years), suggesting that other metabolic factors might play a role in this association [8]. On the other hand, hypertension is more prevalent in periodontitis patients who also present with diabetes and obesity, likely due to shared inflammatory and metabolic pathways. These findings support the need for integrated care addressing both oral and systemic health. Furthermore, recent evidence supports the role of periodontal therapy in regulating BP which is likely due to improved endothelial function and decreased systemic inflammation [9]. Lifestyle interventions, including dietary modifications, regular physical activity and smoking cessation, may help mitigate risk factors contributing to both diseases. Given the strong association between periodontitis and hypertension, multidisciplinary approaches integrating periodontal care into cardiovascular risk management should be further explored through larger clinical trials and health-economic evaluations. Although this study improved the understanding of the association between periodontitis and hypertension, the sample size was relatively small, which may limit generalizability. Additionally, the cross-sectional nature of the study precludes establishing causation. Future longitudinal studies with larger cohorts and better control of confounding variables are warranted to further elucidate the causal relationship between hypertension and periodontitis.

## Conclusion:

We show hypertension as a potential risk factor for periodontitis, with systemic inflammation likely acting as a key mediator. Understanding this interplay is important for developing integrated healthcare strategies aimed at effective management of both conditions. Collaborative efforts between dental and medical professionals may improve patient outcomes and support a multidisciplinary approach to chronic disease management.

## Advancement to knowledge:

The present study provides region-specific epidemiological evidence from the Indian population, where both hypertension and chronic periodontitis are highly prevalent but well-adjusted analytical data remain limited. It clarifies whether hypertension acts as an independent risk factor for periodontitis, whether the association is driven by shared metabolic and systemic determinants, or whether the relationship is bidirectional. Assessing the prevalence and stages of hypertension among individuals with and without chronic periodontitis further adds depth beyond a simple presence-absence comparison by enabling evaluation of severity gradients. Future longitudinal and interventional studies incorporating inflammatory and vascular biomarkers, along with multi-center research across diverse Indian populations, are needed to establish causality and support integrated medical-dental preventive strategies.
